# Delays in seeking an abortion until the second trimester: a qualitative study in South Africa

**DOI:** 10.1186/1742-4755-4-7

**Published:** 2007-09-20

**Authors:** Jane Harries, Phyllis Orner, Mosotho Gabriel, Ellen Mitchell

**Affiliations:** 1 Women’s Health Research Unit, School of Public Health and Family Medicine, Faculty of Health Sciences, University of Cape Town, Anzio Road, Observatory, 7925, Cape Town, South Africa; 2 Ipas South Africa , P.O.Box 2155, Parklands, 2121, South Africa; 3 Ipas, P.O. Box 5027, Chapel Hill, NC 27514, USA

## Abstract

**Background:**

Despite changes to the South African abortion legislation in 1996, barriers to women accessing abortions still exist. Second trimester abortions, an inherently more risky procedure, continue to be 20% of all abortions. Understanding the reasons why women delay seeking an abortion until the second trimester is important for informing interventions to reduce the proportion of second trimester abortions in South Africa.

**Methods:**

Qualitative research methods were used to collect data. Twenty-seven in-depth interviews were conducted in 2006 with women seeking a second trimester abortion at one public sector tertiary hospital and two NGO health care facilities in the greater Cape Town area, South Africa. Data were analysed using a grounded theory approach.

**Results:**

Almost all women described multiple and interrelated factors that influenced the timing of seeking an abortion. Reasons why women delayed seeking an abortion were complex and were linked to changes in personal circumstances often leading to indecision, delays in detecting a pregnancy and health service related barriers that hindered access to abortion services.

**Conclusion:**

Understanding the complex reasons why women delay seeking an abortion until the second trimester can inform health care interventions aimed at reducing the proportion of second trimester abortions in South Africa.

## Background

The South African Choice on Termination of Pregnancy (CTOP) Act, no.92 of 1996, replaced the previously restrictive Abortion and Sterilization Act, no.2 of 1975. The Act promotes a woman's reproductive right and choice to have an early, safe and legal abortion [1]. As a direct result of the new abortion legislation, abortion related morbidity and mortality have decreased significantly [2]. However, despite the legislation there are still major barriers to women accessing abortion services. These include provider opposition, stigma associated with abortion, poor knowledge of abortion legislation, a lack of providers trained to perform abortions and facilities designated to provide abortion services particularly in the rural areas [3-6].

Abortion is a time-restricted health service. The CTOP Act provides for abortion on request up to and including 12 weeks of gestation. In cases of socio-economic hardship, rape, incest and for reasons related to the health of the pregnant woman or foetus, terminations can be performed up to 20 weeks of gestation. From 20 weeks onward terminations are available under very limited circumstances. All abortions are performed at facilities designated by the National and Provincial Departments of Health. The CTOP Act allows for first trimester abortions (up to 12 weeks gestation) to be performed by trained nurse midwives, whereas second trimester abortions are provided by doctors.

In recent years the number of abortions performed nationally and in each of the provinces, including the Western Cape has increased substantially, indicating increased availability and accessibility to TOP services [7]. Second trimester abortions account for over 20% of abortions performed in South Africa [7], which is greater than other countries with legalized abortion such as the United States and Vietnam, where 12% or less of abortions occur in the second trimester [8,9]. Abortions performed after 12 weeks of gestation pose greater risks of medical complications than abortions performed during the first trimester [9,10].

While the availability of second trimester abortion services is an important aspect of reproductive health care, reducing its proportion has several public health care advantages. These advantages include decreased risk of procedure related complications, decreased cost to the health services and increased feasibility of abortions becoming a predominantly primary health care service [9,10].

Understanding reasons for abortion delay may facilitate the improvement of referral networks and promote the development of health education programmes that reduce the need for second trimester abortions [11,12]. Yet, despite the need for interventions to encourage earlier abortions, it is important to ensure that second trimester abortion services continue to be accessible and available to all women in South Africa.

In South Africa, little is known about the reasons why women delay seeking an abortion until the second trimester [13]. Exploring the reasons why women attend later in pregnancy for an abortion is important for informing interventions to reduce the proportion of second trimester abortions in South Africa. This paper reports on results from a qualitative study that explored the factors that contributed towards women accessing abortion services in their second trimester of pregnancy.

## Methods

### Study sites

The study was conducted over a four month period in 2006 at a public sector tertiary hospital and two non-governmental organization (NGO) health care clinics located in the greater Cape Town area, Western Cape, South Africa. All three study facilities provide both first and second trimester abortion services and are broadly representative of the types of services available for women seeking a second trimester abortion in the Western Cape Province of South Africa. Services at the public hospital are provided free of charge whereas payments for services are required at the NGO facilities. These three facilities were selected as they all provide second trimester services using the dilation and evacuation (D&E) method. In the Western Cape Province, most second trimester abortions performed in the public and NGO sector use the D & E method. A few tertiary hospitals provide the medication method of abortion using misoprostol-alone regime which requires several days of hospital admission. These hospitals were not considered suitable for several reasons: the small numbers of second trimester abortions performed at these facilities made recruitment difficult, and, in addition, interviewing women while they were aborting, in discomfort and with limited privacy, was not deemed appropriate.

### Study Respondents

A total of 27 in-depth interviews were conducted with women seeking a second trimester abortion. Participants were selected through purposive sampling. Interviews were conducted either on the day of the abortion prior to the procedure or on the booking day according to the participant's preference. Misoprostol 400 – 800 micrograms was administered orally to all women prior to the D& E procedure to facilitate cervical ripening. Women at all three sites initially made a booking and then returned on a pre-scheduled day for the abortion. Gestational age was confirmed by ultrasound at the booking visit.

The study interviewers visited the three facilities on the days that women either made an appointment or on the day of the abortion. Women presenting for a second trimester abortion were identified by the facility staff according to the gestational age determined by ultrasound. These women were then approached by the interviewers and invited to participate in the study. Out of the 39 women approached, 12 refused to participate citing the need for privacy and/or physical and emotional discomfort as reasons for not participating.

### Study design

In-depth interviews were undertaken with women seeking a second trimester abortion. Individual in-depth interviews were deemed more appropriate for women seeking an abortion due to the sensitivity of the subject matter and the need for privacy.

Interview guides were semi-structured, open-ended and probing. Key issues explored included: barriers to accessing abortion services including both personal and structural barriers; knowledge of the CTOP Act and the circumstances under which a woman can obtain an abortion; recognition and response to an unplanned pregnancy and contraceptive practices.

Interview guides and consent forms were piloted to check for language appropriateness and understanding. Experienced fieldworkers trained in qualitative research methods conducted the in-depth interviews in the participant's first language Xhosa, or English. Interviews were tape-recorded and transcribed verbatim. All participants provided written informed consent. Confidentiality and anonymity were ensured. Ethical approval was obtained from the Research Ethics Committee, Faculty of Health Sciences at the University of Cape Town. Approval to conduct the study was obtained from the Western Cape Provincial Department of Health and from the two NGO clinics.

### Data analysis

Data were analysed using a grounded theory approach, based on a process that helps researchers to "discover" categories, themes and patterns that emerge from the data. Initial categories for analysing data were drawn from the interview guide and themes and patterns emerged after reviewing the data within and across respondent groups. The data was coded manually by two senior members of the research team. Trends and crosscutting themes were identified and issues for further exploration were prioritized for final analysis. Any coding discrepancies were resolved through discussion and consensus between research group members.

## Results

Reasons why women delayed seeking an abortion were complex and often interrelated and in turn affected the timing when the abortion was carried out. Broad categories to emerge for reasons for experiencing delays in obtaining an abortion were linked to personal circumstances leading to uncertainty and indecision, difficulties in detecting a pregnancy in the first trimester, and health service related barriers that hindered access to TOP services. Almost all women described multiple and interrelated factors that influenced the timing of seeking an abortion.

### Socio demographic characteristics

Socio-demographic information was collected from all 27 respondents prior to the interview. The mean age of respondents was 26.8 years (range 16–40), all women had secondary school education, 18(67%) were in a relationship and 4 women (15%) were unemployed. Eighteen (66%) respondents had had previous pregnancies with one woman reporting a prior abortion. Two (7.4%) of respondents reported using a hormonal contraceptive method at the time of conception. The average gestational age at booking was 16.8 weeks (range14 – 19.6) with no differences in terms of gestational age for women presenting at public sector and NGO facilities.

### Pregnancy recognition, confirmation and response

Many participants recalled "signs" and symptoms of pregnancy such as tender breasts, certain food "cravings" and nausea, but did not initially relate these symptoms to a possible pregnancy. Moreover, many women reported a history of irregular periods and often waited until the second or third month of a missed period before seeking pregnancy confirmation suggesting that a missed period was not linked to a suspected pregnancy.

Women with a history of irregular periods explained how they had waited until the second or third month of a missed period before confirming their pregnancy. One woman explained:

'I was in my third month, but because I have problems with my monthly period, I just took it, okay, maybe it's one of those months where it's not going to come. And then, when I didn't get it in the third month, I went to the chemist and I bought a self pregnancy test and I tested and it came out positive... But like I said, because my period is on and off, sometimes I get it, sometimes I won't get it. I just took it, okay, maybe it's going to come the next month.'

Almost all women confirmed their pregnancy by administering a home pregnancy test which they bought privately from a pharmacy or drugstore, and whilst experiencing difficulties in initially suspecting a pregnancy, were cognizant of ways to independently confirm a pregnancy.

A woman who typically experienced irregular periods coupled with having had no pregnancy symptoms did not "realize" that she was pregnant. On discovering she was pregnant she waited a further four weeks before deciding to have an abortion as she was "still in doubt". Reflecting on the complex process from suspecting a pregnancy to seeking an abortion, she explained:

'It's something normal that my periods are on and off, sometimes I have, sometimes I won't. You just try to convince yourself that you are not pregnant. But at the end of the day you have to go and take the [pregnancy] test...Maybe it happens that you don't want to admit your responsibility I don't know.'

Participants responded in diverse ways to an unplanned pregnancy ranging from "shock" and disbelief to being "upset and sad" and not "ready to have a child". Whilst some women were initially "happy to be pregnant", upon reflection of their personal and financial circumstances reconsidered their options.

### Contraceptive use

Most women interviewed were not using a contraceptive method. The relationship between non contraceptive use and linking a missed period to a possible pregnancy appeared somewhat elusive and for many, the situation was further compounded by a history of irregular menses. Women were either not using a contraceptive method or reported contraceptive failure. Establishing why women were not consistently using contraceptives was difficult. Reasons mentioned were "forgetting to take the pill", difficulties associated with injectable contraceptives such as weight gain and irregular menses, partner dislike of condoms and not being in a relationship. Explained by a young woman:

'I saw that there was no need for me using contraceptives because I didn't have a man and I told myself that I must stop using contraceptives, then I had a man and I forgot it.'

It would appear that the two respondents who had used contraception, however inconsistently, thought they would be protected which might have prevented them from identifying their pregnancy earlier.

### Seeking an abortion: personal and emotional factors

#### Personal

Ambivalence and uncertainty often led to delays in seeking an abortion. Most women indicated that they were not currently able to have a child based on their personal and social circumstances. Some women said they were not "ready" or financially able to have a child, whereas others wanted to continue with schooling and studies or were motivated by partner discord.

A woman with a four year old daughter who wanted two more children before the age of 30 was initially relieved to be pregnant but changed her mind later on.

'When I discovered it ... it was sort of a relief because I was pregnant before 30, so it's like I'm looking forward to having a baby because I love babies. And then when you accept it like that, and then Boom! Then the circumstances, that's when everything just starts to come in... You just look at yourself, you don't have a permanent home, you don't have a permanent job... and then you start looking at all those things. It's like at the end of the day you tell yourself, okay, I understand I need a baby but there's no time for it. There is no time.'

A woman with tuberculosis and newly diagnosed with HIV/AIDS was clear in her intent to have an abortion due to her compromised health status and the need to bring up her young child with limited financial means but still felt that "God would punish [her] for seeking an abortion".

'I can't go through like this because I've got other things on my mind. I've got other problems to sort out, you know, through my life, because, it's my health now and so I decided no, I have to do this because I must think of myself, my health, and my son who needs me because my son's father doesn't help me.'

Family, friends and partners played a role in women's decisions around either continuing or terminating a pregnancy. Responses ranged from support to being afraid to discuss their intention to terminate a pregnancy.

Some women were able to discuss their decision to have an abortion with their boyfriends, yet others were afraid to discuss their decision because of fear of being abandoned.

'I went back and told my boyfriend I was pregnant and we discussed it and made a decision no, I don't want this baby. He already has two kids from a previous relationship and he's in a divorce situation... and that's already not a very nice situation so I said I didn't want to. He was going through some stress of his own, but obviously I made it quite clear that it's my choice.'

Another respondent described how she was unable to tell her boyfriend about having an abortion but was supported by both her mother and a close friend.

'I didn't tell my partner about coming for an abortion, because he wants a baby and I don't want a baby at this time, so I didn't tell him...I told one of my friends...she said, I can't tell you what to do, but if you feel it's the right thing to do then you must do it... She supports me because she knows my reason. I also told my mother where I am going and what I 'm going to do. She didn't shout at me because she knows that I have a child and I'm not going to afford another one because I'm not permanently at work, I'm still on a contract.'

#### Emotional

Emotional responses to an unplanned pregnancy such as shame, fear and indecision were contributing factors in delaying access to abortion services. Whilst some women were clear in their intent to have an abortion others felt conflicted and undecided. Women spoke about being "scared and afraid" and not knowing what to expect in terms of the procedure and anticipated pain. A woman described having "sleepless nights" weighing all the possibilities:

'I was hoping ... you just keep on hoping maybe I can get a solution, maybe besides getting an abortion, maybe, I can do something. But then eventually you're just going through it, you know – at the end of the day you just come to think of it, okay, I don't want to give my mum another burden, I don't have to be a burden to someone else.'

Another respondent described the enormous guilt she felt in deciding to have an abortion:

'I didn't want to get up because I feel bad and... I know I'm taking a life – but maybe it's because I feel that I've got no other choice, and at this point in time I won't be able to give the child what is rightfully, his or hers. ... This whole week I didn't feel like going anywhere, just to lock myself up in a room and just let something happen to me because I know that I'm going to be punished for doing what I am going to do today.'

### Knowledge of abortion legislation

All women were aware that abortions are legal in South Africa and of their right to seek an abortion, however, they did not necessarily consider it "right" in terms of their religious, personal or moral beliefs. Apart from one respondent, women had little knowledge of the specific details of the abortion legislation, particularly the difference between a first and second trimester abortion and the time restrictions involved, as this interchange suggests:

Respondent: 'I was aware that it was legal... and that you had to be I think 12 weeks or less, and then I basically took a chance because like I said I knew I was around about 3 months pregnant, which is equal to 12 weeks, and I came here and they explained to me, okay, but you can terminate it up until 20 weeks.'

Interviewer: 'Did you know that before?'

Respondent: 'No, I didn't. I guessed that I was around 12 weeks. I thought maybe I will make it, maybe I won't make it. I came here anyway and that was how I found out that they can do it up to 20 weeks.'

Women reported limited prior knowledge of where to obtain an abortion and employed social networks such as family and friends to find out about it. Information was also obtained through print media and through the radio, television and abortion related websites.

### Difficulties accessing abortion services

In addition to the personal factors mentioned above, participants raised problems related to accessing abortion providing facilities, particularly in the public health sector, leading to further delays in receiving an abortion.

#### Staff attitudes

Many women spoke about negative and judgmental attitudes displayed by staff at public health facilities and related instances where staff were not only rude and hostile but attempted to dissuade them from having an abortion. A respondent described how providers imposed their own religious beliefs by bringing out the Bible during a consultation, resulting in her obtaining an abortion at another facility. This was further underscored by structural barriers ranging from inappropriate referrals and being sent from one facility to another before being seen, to waiting a further two weeks for an appointment as clinics were fully booked.

A woman described how she had visited four facilities and one doctor twice before finally being assisted in obtaining an abortion and by this stage was in her second trimester. All these delays had financial implications as well. For those women who were employed it meant organizing time off work and transport costs incurred by being sent from one facility to another.

Negative perceptions of public sector facilities led some women to seek help within the private sector, which not only had financial implications but also meant a delay in obtaining an abortion as women had to save enough money first.

Moralising and judgmental attitudes displayed by providers made it difficult for women, by creating further discomfort.

'You feel like they are just looking at you, they're just looking at someone who is cheapskate, who doesn't have any morals. Someone who thinks, okay, fine, I'm going to fall pregnant today, tomorrow I'm going to take it out. But what they don't understand is – it's not that simple to come to that kind of a decision. That's the most difficult thing.'

However, not all experiences were negative and some women spoke about friendly and helpful staff that assisted in facilitating the process of obtaining an abortion in both the public and NGO facilities.

### Abortion and stigma

Women mentioned that even though abortion was legal in South Africa, it was still stigmatized and not a subject discussed openly in their communities. Some women were reluctant to visit health care providers or clinics within their communities for fear of being recognized and ostracized, consequently impacting on their right to choose.

A woman who chose to obtain an abortion outside of her residential area was still concerned about being seen and described how she was disturbed by anti-abortion demonstrators outside of the clinic. She explained:

'They [demonstrators] totally freaked me out...I looked away, but I told myself that they said something about you know, having a baby is best, whatever, but I thought but I've got my baby, so really, please give me a break. I know ... morally, some people think it's wrong.'

A respondent compared the stigma associated with HIV/AIDS to the stigma associated with having an abortion, suggesting that choice may be double-edged.

'They reject that person because this thing has a stigma like AIDS...because it's a disease that people talk about a lot. They forget that maybe you have a choice and it's up to you ... whether you can do it or not...and people don't want to mix with you if you have had an abortion.'

### Religious influences

Most women spontaneously related to their own religious beliefs and how this impacted on decisions to seek an abortion. While they subscribed to religious norms that depicted abortion as "murder" or an "abomination," an unwanted pregnancy placed them in a quandary. Shame and guilt associated with seeking an abortion was often eased by prayer. A highly religious woman, faced with either making a choice or disappointing her family, sought approval through prayer.

'All I'm going to do is pray. I'm still praying with God and I still ask him, is that the right thing I'm doing. ... I've asked him show me the way, don't let somebody tell me the way, you show me the way, and I believe in God, he's going to show me the way. He gives people knowledge and he said use your knowledge on the right time. I think it's for me the right time to use it, do my own will.'

Yet for many women, having an abortion was ultimately a private matter, illustrated by one woman's comment that although "it wasn't right to have an abortion it remained a personal matter between me and my God".

## Discussion

The complex decision making process women with an unintended pregnancy are faced with impacted on timely access to abortion services. Women were undecided in their decision to terminate a pregnancy which led to delays in seeking an abortion. These delays were further compounded by health service related barriers such as inappropriate referrals and long waiting-periods.

Similar to other studies, our study findings suggest that problems in suspecting a pregnancy were an important cause of delay [11,12,14]. Reported problems with irregular periods and poor recall and recording of menses, resulted in difficulties recognizing pregnancy symptoms, which, if identified earlier, may have prompted women to confirm a pregnancy sooner.

Overall poor contraceptive uptake amongst study participants in a relatively well resourced urban area of South Africa is cause for concern. South Africa has a relatively high contraceptive prevalence (61%) compared to other sub-Saharan African countries with the Western Cape Province having better overall reproductive health care services than most other areas of South Africa[15].

Despite limited or no contraceptive use, women did not make the link between amenorrhea and a possible pregnancy. On the one hand, women experienced difficulties in detecting a pregnancy with at least two months elapsing prior to pregnancy confirmation. Yet on the other hand, they were prompt in confirming a pregnancy through independent means by administering a home pregnancy test. Attention to one aspect of reproductive health and not to another would require further exploration. It is interesting to note that women did not access pregnancy testing services within the public sector. The latter is not surprising. A recent study in a similar setting suggested poor utilization of urine pregnancy testing in public sector clinics [13].

Whilst women knew that abortion was legal, most were not aware of the time restrictions involved [6], suggesting that merely knowing when, where and who can request an abortion is not enough. Information on the availability of abortion services particularly the time restrictions involved should be included in reproductive health care counselling so that women with unintended pregnancies are able to make informed choices.

Most women described multiple barriers to obtaining an abortion before the second trimester and did not necessarily identify one reason as being more important than another. Women tended to relate more to social and personal issues than service related barriers. However, with further probing it did become clear that many women had reservations about judgmental and negative attitudes displayed by providers at public sector facilities and overall concerns about being further stigmatized for seeking an abortion.

This study revealed several important shortcomings in the health care system and with regards to abortion care provision. Initial delays in suspecting a pregnancy was underscored by further delays once women decided to have an abortion. Delays due to inappropriate referrals evidenced by women attending numerous facilities before obtaining an abortion, waiting periods of over two weeks and difficulties locating a facility providing second trimester abortions is concerning. Women's access to abortion care services is further compromised by general resistance of health care workers to provide second trimester abortion care [5,16]. As a result, second trimester abortion services are being discontinued at some public health care facilities [5].

Women intimated that reproductive choice was often difficult, particularly in a climate of judgmental and negative attitudes displayed by healthcare providers. Antipathy and staff rudeness is not an unexpected issue to emerge and has been reported in South Africa within both family planning and abortion care services [4,5,17]. This situation underscores the need to destigmatize abortion. Opportunities for values clarification training designed to promote more tolerant attitudes by service providers should continue and extend to health care providers working within all areas of reproductive health. Such interventions would play an important role in improving the quality of care and long term health outcomes of women seeking an abortion. The possibility of NGO's supporting the public sector in providing client centred, supportive abortion care services in areas where TOP services are not available should be further explored.

Partners, family and friends played an important role in decisions whether to have an abortion, yet women independently emphasized their own decision-making autonomy. Religious beliefs could have been a contributing factor in delaying seeking an abortion. Fear of being ostracized from the church, family and wider community and difficulties reconciling religious beliefs around the sanctity of life with "taking a life" were underscored by the realities of women's daily lives and personal circumstances.

### Limitations

This study has possible limitations. Despite efforts to provide a non threatening environment, respondents may have experienced difficulties discussing sensitive information and finding out about the actual reasons for waiting until the second trimester of pregnancy was often difficult. Moreover, the findings reflect the circumstances affecting a particular population, i.e., women located within an urban area with better health care infrastructure and more designated abortion facilities than women located within the rural areas of South Africa. We also did not explore whether there were differences between women seeking abortion services earlier compared to those seeking a second trimester abortion [13] but focused more on the circumstances.

## Conclusion

To our knowledge, this is the first qualitative study undertaken in South Africa exploring the reasons why women delay seeking an abortion until the second trimester. This adds to the body of information addressing the barriers to safe abortion services since the abortion legislation was passed in 1996 [3-6].

A complex set of personal circumstances coupled with structural barriers led women to delay seeking an abortion until the second trimester of pregnancy. Several public health measures may decrease the proportion of second trimester abortions. In addition to improving women's access to contraceptive counselling, including post abortion contraceptive counselling, efforts should be made to improve women's knowledge around pregnancy, reproductive physiology and reproductive health in general. Furthermore, women should be educated about the importance of maintaining menstrual records or diaries. Health care professionals should be encouraged to provide women with comprehensive information before they become pregnant as well as facilitating timely referrals and decision-making after pregnancy has been diagnosed. However, due to the personal and complex nature of many of the reasons for delay, the need for legal and accessible second trimester abortion services will remain necessary to provide safe abortion care services.

## Competing interests

The author(s) declare that they have no competing interests.

## Authors' contributions

JH conceptualized and designed the study, oversaw data collection and conducted data analysis and drafted the manuscript.

PO assisted in designing the study, data collection and analysis and reviewed the manuscript.

MG and EM reviewed the manuscript.

All authors read and approved the final manuscript.

## References

[B1] South African Parliament Choice on Termination of Pregnancy Act, Act no.92 of 1996.

[B2] Jewkes R, Rees H (2005). Dramatic decline in abortion related mortality due to the Choice on Termination of Pregnancy Act. S Afr Med J.

[B3] Harrison A, Montgomery ET, Lurie M, Wilkinson D (2000). Barriers to implementing South Africa's Termination of Pregnancy Act in rural KwaZulu/Natal. Health Policy Plan.

[B4] Jewkes R, Gumede T, Westaway M, Dickson K, Brown H, Rees H (2005). Why are women still aborting outside designated facilities in metropolitan South Africa?. BJOG.

[B5] Varkey SJ (2000). Abortion services in South Africa: available yet not accessible to all. Int Fam Plan Perspect.

[B6] Morroni C, Myer L, Tibazarwa K (2006). Knowledge of the abortion legislation among South African women: a cross-sectional study. Reproductive Health.

[B7] South African Department of Health (2005). Termination of Pregnancy Update Cumulative Statistics through 2004.

[B8] Strauss L, Herndon J, Chang J, Parker W, Bowens S, Berg C (2004). Abortion surveillance:United States,2001. MMWR Surveill Summ.

[B9] World Health Organization (2004). Safe Abortion: Technical and Policy Guidance for Health Systems.

[B10] Bartlett L, Berg C, Shulman H, Zane S, Green C, Whitebread S, Atrash H (2004). Risk factors for legal induced abortion-related mortality in the United States. Obstet Gynecol.

[B11] Drey E, Foster D, Jackson R, Lee S, Cardenas L, Darney P (2006). Risk factors associated with presenting for abortion in the second trimester. Obstet Gynecol.

[B12] Finer L, Frohwirth L, Dauphinee L, Singh S, Moore A (2006). Timing of steps and reasons for delays in obtaining abortions in the United States. Contraception.

[B13] Morroni C, Moodley J (2006). Characteristics of women booking for first and second trimester abortions at pubic sector clinics in Cape Town. SAJOG.

[B14] Cooper D, Dickson K, Blanchard K, Cullingworth L, Mavimbela N, von Mollendorf C, van Bogaert L, Winikoff B (2005). Medical abortion: The possibilities for introduction in the public sector in South Africa. Reprod Health Matters.

[B15] Department of Health, Republic of South Africa (2001). South African Demographic and Health Survey. Pretoria Department of Health.

[B16] Mitchell E, Trueman K, Gabriel M, Bock L (2005). Building alliances from ambivalence: evaluation of abortion values clarification workshops with stakeholders in South Africa. Afr J Reprod Health.

[B17] Wood K, Jewkes R (2006). Blood blockages and scolding nurses: barriers to adolescent contraceptive use in South Africa. Reprod Health Matters.

